# Hirshfeld atom like refinement with alternative electron density partitions

**DOI:** 10.1107/S2052252520013603

**Published:** 2020-10-29

**Authors:** Michał Leszek Chodkiewicz, Magdalena Woińska, Krzysztof Woźniak

**Affiliations:** a Biological and Chemical Research Centre, Department of Chemistry, University of Warsaw, Żwirki i Wigury 101, Warszawa, 02-089 Warszawa, Poland

**Keywords:** Hirshfeld atom refinement, electron density partition, generalized atom refinement, GAR, HAR

## Abstract

Hirshfeld atom refinement is one of the most successful methods for the accurate determination of structural parameters for hydrogen atoms from X-ray diffraction data. This work introduces a generalization of the method [generalized atom refinement (GAR)], consisting of the application of various methods of partitioning electron density into atomic contributions. These were tested on three organic structures using the following partitions: Hirshfeld, iterative Hirshfeld, iterative stockholder, minimal basis iterative stockholder and Becke. The effects of partition choice were also compared with those caused by other factors such as quantum chemical methodology, basis set, representation of the crystal field and a combination of these factors. The differences between the partitions were small in terms of *R* factor (*e.g.* much smaller than for refinements with different quantum chemistry methods, *i.e.* Hartree–Fock and coupled cluster) and therefore no single partition was clearly the best in terms of experimental data reconstruction. In the case of structural parameters the differences between the partitions are comparable to those related to the choice of other factors. We have observed the systematic effects of the partition choice on bond lengths and ADP values of polar hydrogen atoms. The bond lengths were also systematically influenced by the choice of electron density calculation methodology. This suggests that GAR-derived structural parameters could be systematically improved by selecting an optimal combination of the partition and quantum chemistry method. The results of the refinements were compared with those of neutron diffraction experiments. This allowed a selection of the most promising partition methods for further optimization of GAR settings, namely the Hirshfeld, iterative stockholder and minimal basis iterative stockholder.

## Abstract 2

In this work, various models of atomic electron density were applied in a generalized version of the Hirshfeld atom refinement to three organic structures.

## 1. Introduction   

Ongoing progress in experimental technique development in X-ray crystallography makes this method an excellent tool to observe aspherical electron density deformations that can be attributed to bond formation and other interactions. However, the simplest and most popular approach, in fact, the only one practically available for many decades is the independent atom model (IAM), which treats the crystal as a set of spherical atomic densities centred on the atomic nuclei. However, it does not take into account the aspherical nature of atomic electron densities. For this reason, IAM fails to correctly describe those aspects of molecular geometry which are influenced by aspherical electron density deformations, such as the positions and anisotropic displacement parameters of hydrogen atoms. Consequently, the bond lengths formed by hydrogen atoms are on average shorter by 0.1 Å compared with their benchmark values as reported by neutron diffraction experiments and anisotropic refinements of hydrogen atom thermal motions resulting in non-positive definite ADP values. Therefore, methods introducing the various models of aspherical atomic scattering factors were developed (Weiss, 1964 ▸; DeMarco & Weiss, 1965 ▸; Kurki-Suonio, 1968 ▸; Stewart, 1969 ▸, 1973 ▸; Hirshfeld, 1971 ▸; Hansen & Coppens, 1978 ▸). Unfortunately the most successful and popular of them, the multipole model proposed by Hansen & Coppens (1978 ▸), does not allow for free refinement of hydrogen atom positions and ADP values (Hoser *et al.*, 2009 ▸), except in certain special cases of high-resolution good-quality data (Zhurov *et al.*, 2011 ▸; Woinska *et al.*, 2019 ▸). This problem was overcome by use of the transferable aspherical atom model (TAAM) which takes advantage of the fact that the parameters of the multipole model are similar to those of atoms in similar chemical environments (Pichon-Pesme *et al.*, 1995 ▸) and uses predefined sets of such parameters for refinement. This allows for free refinement of hydrogen positions and leads to more accurate *X*—H bond lengths, as shown previously for a number of databanks of multipole model parameters (Bąk *et al.*, 2011 ▸).

Performing Hirshfeld atom refinement (HAR) (Jayatilaka & Dittrich, 2008 ▸; Capelli *et al.*, 2014 ▸) which utilizes stockholder partitioning (Hirshfeld, 1977 ▸) of the electron density for a molecule in the crystal turned out to be an even more promising method as it avoids the atomic density transferability assumption used in TAAM and the limitations of the electron density model used in multipole formalism (Koritsanszky *et al.*, 2011 ▸). This implements the atomic aspherical structure factors obtained from the Hirshfeld-partitioned electron density of the asymmetric unit/molecule/cluster in the crystal calculated by an iterative procedure with the effects of the crystal environment included via surrounding the central unit by a cluster of electric multipoles. The improved model of the aspherical atomic structure factor resulted in more accurate and precise refinement of the hydrogen atom positions and considerable progress in the refinement of hydrogen ADP values (Capelli *et al.*, 2014 ▸; Woińska *et al.*, 2016 ▸, 2017 ▸; Malaspina *et al.*, 2017 ▸; Orben & Dittrich, 2014 ▸; Dittrich *et al.*, 2017 ▸; Sanjuan-Szklarz *et al.*, 2020 ▸), even for X-ray data of standard resolution. Therefore HAR was added to a group of methods for deriving ADP values for hydrogen atoms, including the ‘TLS+ONIOM’ approach based on *ab initio* calculations (Whitten & Spackman), refinement with the TAAM model (Dittrich *et al.*, 2008 ▸), TLS-based analysis available via the web service *SHADE* (Munshi *et al.*, 2008 ▸) and lattice dynamical models (Hoser & Madsen, 2016 ▸; 2017 ▸).

HAR was applied to refinement of anharmonic thermal motions (Woinska *et al.*, 2019 ▸; Orben & Dittrich, 2014 ▸), refinement of compounds that contain transition metals (Woińska *et al.*, 2016 ▸; Bučinský *et al.*, 2016 ▸, 2019 ▸; Malaspina *et al.*, 2019 ▸) and refinements including relativistic effects (Bučinský *et al.*, 2016 ▸, 2019 ▸; Malaspina *et al.*, 2019 ▸). Initial work aimed at optimizing HAR for the refinement of macromolecules is also available: HAR-ELMO (Malaspina *et al.*, 2019 ▸) and fragHAR (Bergmann *et al.*, 2020 ▸). A recent study of TAAM refinement (K. Jha *et al.*, 2020 ▸) using the same set of test systems, as in an analogous study of HAR (Woińska *et al.*, 2016 ▸) revealed that HAR produced bond lengths slightly closer to those obtained from neutron diffraction than TAAM. The average bond length underestimation was 0.020 Å for TAAM and 0.014 Å for HAR. It should be noted that such results apply for specific TAAM parameterizations and specific HAR methodologies (defined by the quantum chemical method, basis set and representation of crystal field).

Nevertheless, HAR is still not a fully mature method since there are still many areas with potential for improvement including long computational times required for repeated quantum mechanical calculation; quality of hydrogen ADP values refined with HAR; refinement of structures other than those of molecular crystals (network structures, ionic crystals) may be suboptimal; refinement of disorder is not yet properly handled; refinement of structures containing heavy metals is difficult due to the limited choice of available basis sets and challenges related to application of relativistic methods; lack of a well established optimal combination of settings (including the quantum chemistry method, basis set and representation of crystal field).

It must also be stressed that increasing the applicability of HAR, adapting the method to perform more challenging tasks such as refinement of macromolecular structures and increasing its popularity among users requires the creation of new software tools and/or the incorporation of the method in existing, commonly used programs dedicated to the processing of crystallographic data. A step in this direction was its implementation in a popular program for chemical crystallography *OLEX2* (Dolomanov *et al.*, 2009 ▸) in the pre-installed *HARt* interface, enabling simple access to the basic functionalities of HAR (Fugel *et al.*, 2018 ▸). Refinement with *HARt*, similar to the classical version of HAR (Jayatilaka & Dittrich, 2008 ▸; Capelli *et al.*, 2014 ▸) can be carried out against *F*, unlike IAM in *OLEX2*, which is based on *F* ^2^. As far as treatment of macromolecular structures is concerned, the development of HAR-based methods is proceeding in two directions: database-related methods and fragmentation techniques. The first involves the HAR-ELMO method (Malaspina *et al.*, 2019 ▸) which combines HAR with libraries of extremely localized molecular orbitals (Meyer & Genoni, 2018 ▸). This method was tested on a few small-molecule structures and proved capable of locating hydrogen atoms (in terms of bond lengths and ADP values) as accurately and precisely as traditional HAR at significantly lower computational costs. It was also successfully applied in the refinement of two polypeptides and the crystal structure of crambin (for two X-ray datasets collected at different subatomic resolutions). The other fragmentation-related method was first implemented in the fragHAR method (Bergmann *et al.*, 2020 ▸) using molecular fractionation with the conjugate caps method of fragmentation (Zhang & Zhang, 2003 ▸) in order to divide the molecule of interest into smaller fragments for which a wavefunction can be calculated with quantum mechanical methods. This method was implemented in the *TONTO* program (Jayatilaka & Grimwood, 2003 ▸) and tested on three oligopeptide crystal structures. It yields hydrogen positions and ADP values in statistical agreement with HAR; however, any interactions that involve hydrogen atoms must be given special attention during the fragment-selection process.

HAR is based on a model defined by computational chemistry methods (*e.g.* Hartree–Fock or density functional theory with BLYP functional) as well as the basis set and representation of the molecular environment in the crystal. Although there are recommendations to guide the choice of model settings (Capelli *et al.*, 2014 ▸; Fugel *et al.*, 2018 ▸), these are not yet well established. Most HAR refinements have been performed with the Hartree–Fock method or a density functional approach with BLYP functional, which are usually not considered methods of choice for accurate quantum mechanical calculation for molecular systems. Very recently, an application of more accurate quantum chemistry methods [second-order Møller–Plesset perturbation theory (MP2) and coupled cluster singles and doubles (CCSD)] have been tested (Wieduwilt *et al.*, 2020 ▸); however, conclusions about the advantages of these methods over computationally cheaper methods were not discussed. It was observed that an agreement between experimental and HAR-derived structure factors for l-alanine improves in the order HF, BLYP, MP2 ≃ B3LYP, CCSD.

In this study we examine additional options for choosing the model. We test the effects of the choice of electron density partitioning on atomic contributions. Since we are no longer limited to the partition proposed by Hirshfeld, the resulting method can be thought of as a generalization of HAR; to distinguish this from HAR it will be referred to as generalized atom refinement (GAR).

If the effects of partition choice on refinement accuracy are comparably important as those of the other settings in GAR and the newly introduced partitions are not clearly inferior compared with Hirshfeld partition then we can conclude that there is an important new dimension in the search for an optimal HAR-like model. In this work we aim to identify promising directions for such a search.

Partitioning electron density into atomic contributions is closely related to popular chemical concepts such as net atomic charge and atoms in molecules. Many partition methods have been proposed: those related to Bader’s quantum theory of atoms in molecules (Bader, 1990 ▸), to the Mulliken (1955 ▸) and Löwdin (1955 ▸) population analyses (among others), stockholder partitioning as proposed by Hirshfeld (1977 ▸), and related methods such as the iterative Hirshfeld method (Bultinck *et al.*, 2007 ▸), the iterative stockholder partitioning method (Lillestolen & Wheatley, 2008 ▸), the minimal basis iterative stockholder (Lillestolen & Wheatley, 2008 ▸) and the DDEC6 method (Manz & Limas, 2016 ▸). Electron density partitions are also used for computational purposes [*e.g.* in the Becke scheme for numerical integration (Becke, 1988 ▸)]. In this preliminary test of the effect of electron density partitioning on HAR-like refinement, we tested partitions implemented in the quantum chemistry program *HORTON* (Verstraelen *et al.*, 2017 ▸) which mainly includes partitions inspired by the one introduced by Hirshfeld (1977 ▸).

## 2. Methodology   

### 2.1. Investigated structures   

In this work, three organic systems were selected for testing: urea, oxalic acid dihydrate and 9,10-bis-di­phenyl­thio­phospho­ranylanthracene·toluene (SPAnPS). Urea and oxalic acid dihydrate have been used in many studies on accurate refinements with aspherical atom models, and for electron density distributions in crystals (Stevens *et al.*, 1979 ▸; Stevens & Coppens, 1980 ▸; Zobel *et al.*, 1993 ▸; Krijn *et al.*, 1988 ▸; Swaminathan *et al.*, 1984 ▸; Gatti *et al.*, 1994 ▸; Birkedal *et al.*, 2004 ▸; Jayatilaka & Dittrich, 2008 ▸; Pisani *et al.*, 2011 ▸; Wall, 2016 ▸). Urea and oxalic acid are small polar organic molecules, which makes testing them relatively easy with, in general, computationally demanding methods such as GAR. The polarity of these systems is an advantage when examining the differences between the Hirshfeld and iterative Hirshfeld partitions as they are expected to be more pronounced than in non-polar systems. Ionic systems would probably be even more appropriate for such comparison, but HAR methodology for these kinds of systems is not yet well established so was not examined. The third system, SPAnPS, contains substantially larger molecules with no polar hydrogen atoms. Each X-ray dataset has known neutron measurements at the same temperature that we measured. We used the following sources of datasets/structures: urea – neutron structure (Swaminathan *et al.*, 1984 ▸) and X-ray data (Birkedal *et al.*, 2004 ▸) both at 123 K, oxalic acid (Kamiński *et al.*, 2014 ▸), and SPAnPS (Köhler *et al.*, 2019 ▸). ADP values for oxalic acid neutron measurement were scaled isotropically because we noticed systematic differences in ADP values of non-hydrogen atoms. The scale factors were derived by the method of least squares (Blessing, 1995 ▸). One of possible explanations is a difference in true temperatures of diffraction experiments.

### 2.2. Partitions   

The following partitions were examined in this study: the original stockholder partition proposed by Hirshfeld (H) (Hirshfeld, 1977 ▸) which was used in HAR, the iterative Hirshfeld (IH) (Bultinck *et al.*, 2007 ▸), iterative stockholder (IS) (Lillestolen & Wheatley, 2008 ▸), the minimal basis iterative stockholder (MBIS) (Verstraelen *et al.*, 2016 ▸) and the partition proposed by Becke (B) (Becke, 1988 ▸). Most of the tested methods are based on the stockholder partition of the electron density, which expresses the electron density ρ of an atom *a* at point *r* as
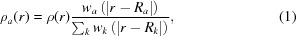
with a summation over all atoms in the system (indexed with subscript *k*), *w_k_*(*r*) is the spherical weighting function for the *k*th atom and *R_k_* is the position of the *k*th atom. Such methods can be viewed as an extension of the original stockholder method proposed by Hirshfeld.

In the original Hirshfeld partition the function *w_k_*(*r*) corresponds to the spherically averaged atomic densities of the isolated atoms. Hirshfeld partitioning leads to relatively low partial charges (Davidson & Chakravorty, 1992 ▸), which has been considered to be a deficiency in the method (Bultinck *et al.*, 2007 ▸). These low partial charges are not surprising since they are maximally similar to the isolated atoms under an information theory framework (Nalewajski & Parr, 2000 ▸).

The iterative Hirshfeld method is similar to Hirshfeld partitioning but also takes the atomic charges into account for the weighting function. The weighting function for a given atom is a combination of the electron densities of the isolated neutral atom and an isolated ion(s) of a given element. This combination is chosen in such a way that it reproduces the charge of the corresponding atom. This method produces considerably higher partial charges than Hirshfeld partitioning which can lead to problems [*e.g.* in the case of one highly polar oxide, calculation of the electron density of a nonexistant oxygen dianion was required (Verstraelen *et al.*, 2013 ▸)].

The iterative stockholder method does not require supplementary atomic density gas-phase calculations. It uses spherically averaged atomic densities for the atoms as weight functions with the initial weight functions normalized to *w_k_*(*r*) = 1 for all atoms. While this method avoids some problems which appear in the Hirshfeld and iterative Hirshfeld methods, it has been shown (Bultinck *et al.*, 2009 ▸) that the spherically averaged atomic densities resulting from this method can be not monotonically decaying, a counterintuitive result.

In the case of the minimal basis iterative stockholder method, the weighting function for a given atom is expressed using a minimal basis set of spherical Slater-type functions. This method is similar to the iterative stockholder method.

The partition proposed by Becke was designed to deal with three-dimensional integration in molecular systems and therefore it is not expected that the resulting atomic charges will correspond to chemical intuition. This partition is not based on stockholder-type partitions. Instead it is similar to Voronoi tessellation with adjustments for atomic sizes and ‘softened’ boundaries (atomic densities are continuous).

### 2.3. Implementation   

A locally modified version of *OLEX2* (Dolomanov *et al.*, 2009 ▸) was used in the refinements. It incorporated a development version of the *DiSCaMB* library (Chodkiewicz *et al.*, 2018 ▸) into the *olex2.refine* module which allows for the application of form factors corresponding to aspherical atomic densities.

In general, GAR implementation is quite similar to HAR implementation (Capelli *et al.*, 2014 ▸; Fugel *et al.*, 2018 ▸). The information necessary for electron density calculation for a non-periodic molecular system representing a given crystal structure is generated by computational chemistry software. This information incorporates either the set of molecular orbitals or the first-order reduced density matrix. The molecular system was built from molecule(s) comprising the asymmetric unit but this can be increased in order to better represent the molecular environment in the crystal. The effect of the crystal field can be modelled by surrounding the studied system with a set of atomic electric multipole moments. Atomic electron densities corresponding to a chosen partition of molecular electron density are calculated and used in the computation of atomic form factors. The form factors are then used in least squares refinement. Since the refinement leads to a new geometry, new quantum chemical calculations are run and a new least squares refinement is performed. This procedure consisting of quantum chemical calculations followed by least squares refinement is repeated until convergence criteria are met.

In practice, our implementation differs not only by allowing more electron density partitions, but also on many other points, among them the most important is probably refinement against 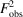. Quantum chemical calculations are performed with *GAUSSIAN16* (Frisch *et al.*, 2016 ▸). The atomic multipole moments needed to represent the effect of the crystal field are calculated using the Hirshfeld partition, even if the atomic form factors are calculated for the other partitions. In this way the equal treatment of crystal field effects for refinements based on different partitions is preserved. Atomic multipoles are calculated in a self-consistent embedding scheme in which a newly calculated electron density is the source for new multipole moments which generate new representations of the crystal field, giving rise to new electron density results. Cycles of such calculations are performed until the differences between the components of the multipole moments are smaller than 0.003 a.u. Only point charges and dipoles are used. Charges representing dipoles are separated by 0.02 Å.

The electron densites at molecular integration grid points were calculated with *HORTON* (Verstraelen *et al.*, 2017 ▸), which reads in the first-order reduced density matrix from a Gaussian formatted check-point file. It also performs molecular electron density partitioning and prints out the atomic electron densities and the details of the molecular integration grid. This is implemented as a part of the *DiSCaMB* library which is responsible for the calculation of atomic multipoles and atomic form factors.

A Becke-type multicenter integration scheme for molecular integrals (Becke, 1988 ▸) is used with pruned grids as defined in *HORTON* (Verstraelen *et al.*, 2017 ▸). A radial grid is generated using the power transform (*r* = *ax* ^*p*^) and a Lebedev–Laikov grid (Lebedev & Laikov, 1999 ▸) is used for angular integration. Form factor calculation involves the largest predefined grid in *HORTON*. Parameters of the grid are element specific, *e.g.* for carbon atoms 148 radial points are used and up to 1730 angular points.

Least squares refinement is performed against 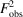 as implemented in *olex2.refine* (Bourhis *et al.*, 2015 ▸) with no use of additional *SHELX*-type parameters in the weighting scheme, *i.e.* the weights are defined as 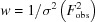, where 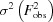 is the variance of the observed intensity. Absence of additional parameters in the weighting scheme allows for direct comparison of the discrepancy in *R* factors. The whole GAR procedure is finished when the difference in geometry after the least squares refinement and the one used in the quantum chemical calculations is less than 0.001 Å for both the atomic positions and the covalent bond lengths.

### 2.4. Reported statistics   

In order to statistically assess the results of GAR refinements, we have compared discrepancy *R* factor values, the lengths of covalent bonds to hydrogen atoms and anisotropic displacement parameters for the hydrogen atoms. The difference between the values obtained from X-ray and neutron measurements are referred to as Δ*R* for bond lengths and Δ*U*_*ij*_ for ADP tensor components. Their average absolute values are calculated (〈|Δ*R*|〉 and 〈|Δ*U*_*ij*_|〉, respectively) and also averaged (〈Δ*R*〉) in the case of Δ*R*. We also calculated the average ratio of the square of the difference to its variance (we reported the root of that value) referred to as the weighted root mean square difference for the bond lengths, wRSMD(Δ*R*), and for the ADP values, wRSMD(Δ*U*_*ij*_). The statistics are defined as follows,
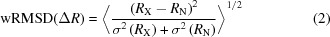
and
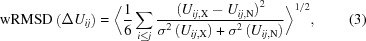
where the subscript X indicates X-ray values, N the neutron values while the angle brackets (chevrons) denote the average value of the expression in the brackets (averaged over atoms). It should be noted that the lower value of wRSMD is not an indicator that one method is better than another since it can happen that a method with higher accuracy and precision corresponds also to higher wRSMD, an example is provided further in the text where the effects of electron density partition choice on ADP values are discussed. We also used the average values of the *S*_12_ similarity index as introduced by Whitten & Spackman (2006 ▸). It is defined as *S*_12_ = 100(1 − *R*_12_), where *R*_12_ describes the overlap between the density distribution functions (*p*_1_, *p*_2_) for nuclei defined by two ADP tensors:
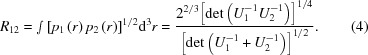
In order to identify patterns in bond lengths, the average ratio of the X-ray to neutron bond lengths 〈*R*_X_/*R*_N_〉 was calculated. In order to identify patterns in the ADP values, we compared the averaged ratios of the volumes of ‘vibrational’ ellipsoids 〈*V*_X_/*V*_N_〉 [*e.g.* known from *ORTEP* (Johnson, 1965 ▸)]. This ratio was calculated by taking into account: (1) the volume of the ellipsoid is proportional to the product of the lengths of its semi-axes, (2) the semi-axes of the thermal ellipsoids are proportional to the eigenvalues of the ADP tensor in Cartesian coordinates and (3) the product of the matrix eigenvalues is equal to its determinant. Taking (1)–(3) together gives
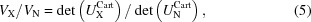
where 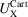 and 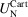 are the X-ray and neutron measurement-based ADP tensors in the Cartesian coordinates, respectively.

When the average over the atoms or bonds is reported, the uncertainty indicated in brackets is given as the population standard deviation, defined as
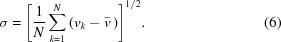



## 3. Results   

In order to compare the results of the GAR refinements with the different partitioning schemes, we focused on the structural parameters related to the hydrogen atoms, specifically the lengths of bonds involving hydrogen atoms and hydrogen ADP values, since accurate determination of these with X-ray refinement is more challenging than for heavier atoms. Unless stated otherwise, all results refer to those parameters.

In order to put the analyzed differences between the experimentally derived X-ray and neutron bond lengths (Δ*R*) into context here are some related values. For example, the standard deviations for bond lengths from neutron measurements referenced in this work for N—H and O—H bonds lie in the ranges 2–3 and 2.5–6.3 mÅ for HAR refinement (for B3LYP/cc-pVTZ), whereas in the case of C—H bonds in SPAnPS those numbers are 1–5 mÅ for neutron data and 3.4–5.7 mÅ for HAR. For example, *X*—H bond lengths from neutron diffraction data (Allen & Bruno, 2010 ▸) for functional groups are C(*sp* ^3^)—O—H 0.970 (12), C(aryl)—O—H 0.992 (17) and O—C(*sp* ^2^)—O—H 1.018 (22) Å, and the differences between those values are 22, 26 and 48 mÅ. The analogous values for HAR (Woińska *et al.*, 2016 ▸; HAR with BLYP/cc-pVDZ) are (data for maximum resolution) 0.953 (28), 0.965 (32) and 0.983 (35) Å and the differences between the HAR and the neutron values (Δ*R*) for maximum available resolution are −17, −27 and −35 mÅ (however, they are smaller for 0.8 Å resolution: −8, −8, −32 mÅ). In the case of IAM the differences are much larger: −122, −147 and −132 mÅ. Although the average distances for HAR are much closer to neutron ones than those from IAM, there are still considerable differences, comparable to the differences in O—H bond lengths for different functional groups. This can be partially explained by the fact that different sets of molecules were used for evaluation of the averages for HAR and neutron data. On the other hand, the average HAR bond lengths reported by Woińska *et al.* (2016 ▸) seem to be systematically shorter than neutron bond lengths in the case of polar hydrogen atoms. We should however remember that these are shorter for HAR using DFT with the BLYP functional and cc-pVDZ basis set. HAR with Hartree–Fock produces longer bonds for polar hydrogens [see the results in the work by Capelli *et al.* (2014 ▸) or Wieduwilt *et al.* (2020 ▸)] as well as higher *R* factors. The problem of the choice of optimal settings for HAR is still far from exhaustively explored.

### 3.1. Testing factors other than electron density partition   

Before discussing the effects of the electron density partitioning method, we will describe the results of tests involving other components of the model including the quantum chemistry method, the representation of the crystal field and the basis set, thereby allowing a comparison between the effects of the electron density partition and the effects related to the other settings (which were not tested in HAR against 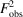). The tests of these factors were performed on urea and oxalic acid since, as relatively small systems, they are well suited for testing computationally demanding settings such as post-Hartree–Fock methods or quantum mechanical representation of molecular surrounding.

These factors were tested with the Hirshfeld partition only. Unless stated otherwise, electron density was calculated using B3LYP, the cc-pVTZ basis set, and the crystal field was represented by atomic point charges and dipoles located at the atoms in molecules with at least one atom within 8 Å of any atom of the molecule for which the wavefunction is calculated. Those settings were selected on the basis of results from the initial phase of refinements for this section. Ideally when testing one of the components of the model, one would use the optimal setting for the other components. This is not possible in practice and not always necessary. At some point, higher levels of theory ceased producing improvements in the results. Little gain has been observed upon switching from the cc-pVTZ to the cc-pVQZ basis set, suggesting that the cc-pVTZ set is more than adequate. In the case of crystal field representation, a model with point charges seemed to produce results of similar quality to those from the more expensive model. B3LYP was selected as the quantum chemical method for the tests since it belonged to the set of methods (B3LYP, MP2, CCSD) which gave the best *R* factors in the initial tests and the two other methods are much more computationally demanding.

**Figure 1:**
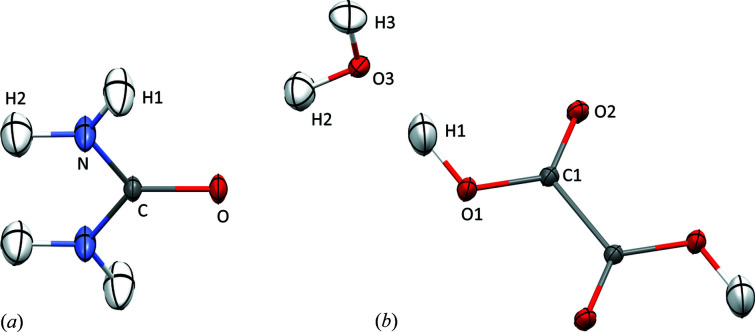
Hydrogen atom labelling schemes for the studied structures (*a*) urea and (*b*) oxalic acid dihydrate, produced using the iterative stockholder partition refinement with B3LYP/cc-pVTZ theory level and surrounding multipoles cluster. ADP values are shown at the 50% probability level (Mercury, Macrae *et al.*, 2020 ▸).

**Figure 2:**
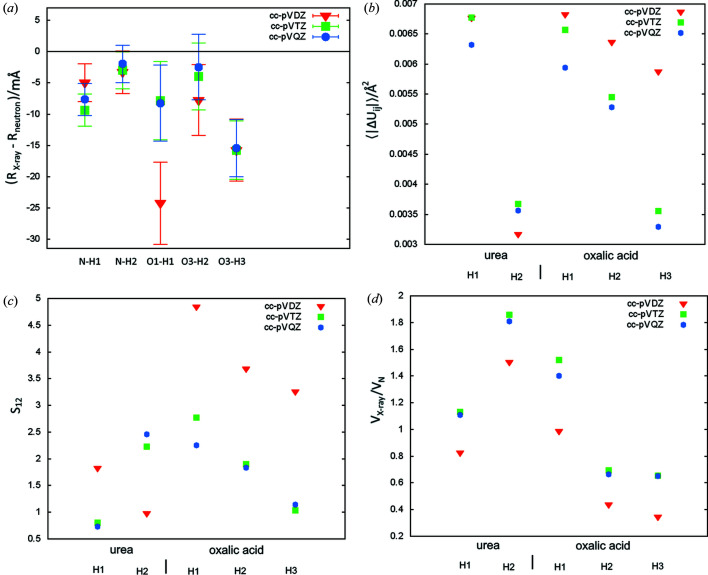
Comparison of neutron and X-ray parameters of polar hydrogen atoms for refinements with various basis sets: (*a*) Δ*R* – the difference between X-ray and neutron measured bond lengths (error bars correspond to the X-ray bond length uncertainties), (*b*) 〈|Δ*U*_*ij*_|〉 – the average absolute difference of the ADP tensor components, (*c*) ADP similarity index *S*_12_, (*d*) *V*_X_/*V*_N_ ratio of X-ray and neutron thermal ellipsoids.

**Figure 3:**
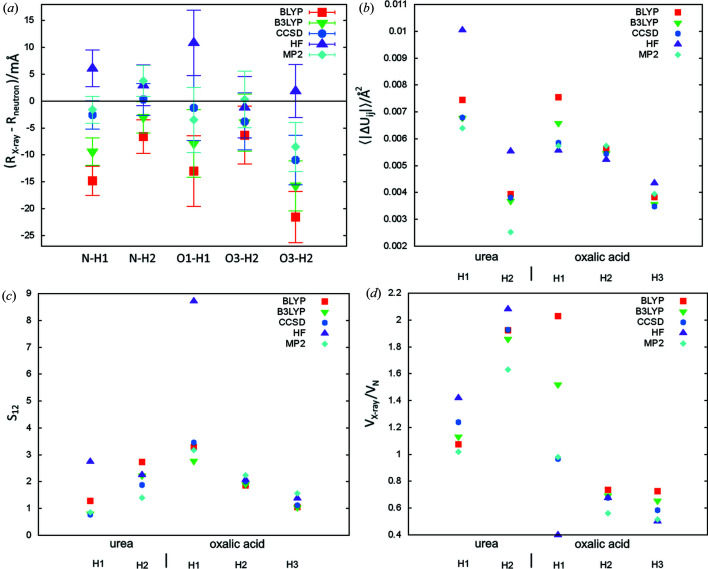
Comparison of the neutron and X-ray parameters of polar hydrogen atoms for refinements with various quantum chemistry methods: (*a*) Δ*R* – the difference between X-ray and neutron measured bond length (error bars correspond to the X-ray bond length uncertainties), (*b*) 〈|Δ*U*_*ij*_|〉 – average absolute difference of ADP tensor components, (*c*) ADP similarity index *S*_12_, (*d*) *V*_X_/*V*_N_ ratio of X-ray and neutron thermal ellipsoids.

**Figure 4:**
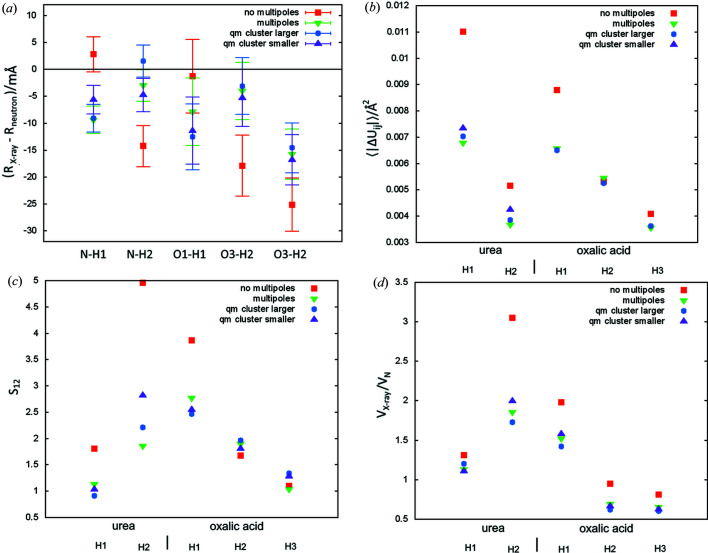
Comparison of the neutron and X-ray parameters of polar hydrogen atoms for refinements with various representations of the crystal field (see text): (*a*) Δ*R* – the difference between X-ray and neutron measured bond lengths (error bars correspond to the X-ray bond length uncertainties), (*b*) 〈|Δ*U*_*ij*_|〉 – the average absolute difference of ADP tensor components, (*c*) ADP similarity index *S*_12_, (*d*) *V*_X_/*V*_N_ ratio of X-ray and neutron thermal ellipsoids.

**Figure 5:**
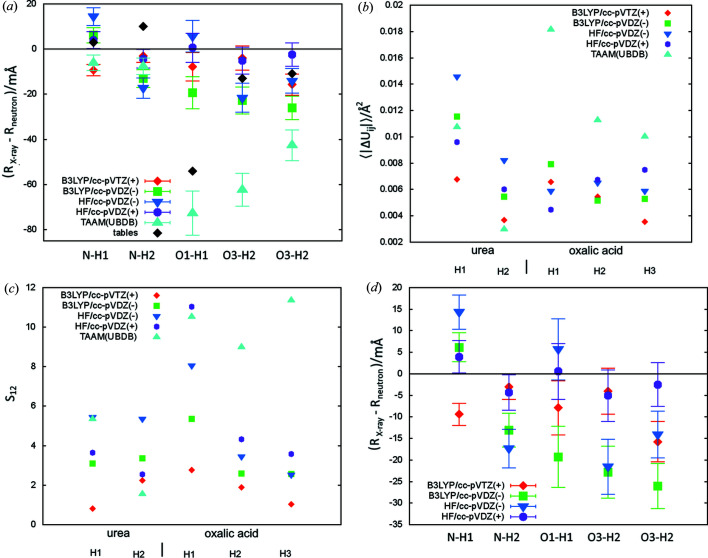
Comparison of neutron and X-ray parameters of polar hydrogen atoms for refinements with HAR, TAAM and a model with standardized neutron bond lengths: (*a*) Δ*R* – the difference between X-ray and neutron measured bond lengths in mÅ (error bars correspond to the X-ray bond length uncertainties), (*b*) 〈|Δ*U*_*ij*_|〉 – the average absolute difference of ADP tensor components, (*c*) ADP similarity index *S*_12_, (*d*) as in (*a*) but the least accurate methods were omitted to improve readability.

The results of the tests are presented in Table 1 ▸. Urea and oxalic acid dehydrate structures have five unique hydrogen atoms (see Fig. 1 ▸), all bonded to electronegative atoms (N and O), referred to throughout the text as polar hydrogen atoms. Values related to the structural descriptors in Table 1 ▸ are given as an average over those atoms. Values of the descriptors are given separately for urea and oxalic acid in Table S1 of the supporting information. Individual values of the structural parameters are shown in Figs. 2–5.

#### 3.1.1. Basis set   

The effect of the choice of basis set on HAR was tested with the family of correlation-consistent basis sets developed by Dunning and coworkers (Dunning, 1989 ▸; Kendall *et al.*, 1992 ▸; Woon & Dunning, 1993 ▸), from the smallest to the largest: cc-pVDZ, cc-pVTZ, cc-pVQZ. Each of the listed basis sets roughly doubles the number of functions of the previous one, making wavefunction calculations considerably slower since computational time formally scales as *N* ^4^ (where *N* is the number of base functions) in the case of the least computationally expensive methods used.

Similar to the earlier tests of HAR (Capelli *et al.*, 2014 ▸), it appears that the cc-pVTZ basis set is sufficient since switching to cc-pVQZ brings only a small reduction in *wR*_2_ [≤0.02 p.p. (percent point)] and relatively small changes in the structural parameters [see Table 1 ▸ and Figs. 2 ▸(*a*)–(*c*)]. Switching from cc-pVDZ to cc-pVTZ leads to a much larger reduction in *wR*_2_ (≤0.3 p.p.). The cc-pVTZ basis set is also visibly better than the cc-pVDZ basis set in terms of the similarity between the X-ray and neutron determined ADP values in the case of oxalic acid [see Figs. 2 ▸(*b*)–2(*c*)], but not in the case of urea. Some patterns can be observed for volumes of thermal ellipsoids: for all five hydrogen atoms those derived with cc-pVDZ are smaller than those obtained with cc-VTZ [Fig. 2 ▸(*d*)]. Discrepancies in bond lengths were especially visible for O1—H1 bond in oxalic acid [Δ*R* 8(6) mÅ for cc-pVTZ and 24 (6) mÅ for cc-pVDZ], which is involved in a very strong hydrogen bond (O1—H1⋯O3, H1 to O3 distance 1.423 Å). Results for the other bonds alone do not suggest that cc-pVTZ is superior to cc-pVDZ for estimating bond lengths. Also the results in the work by Capelli *et al.* (2014 ▸) do not show that the use of a larger basis set (cc-pVTZ) leads to better bond lengths (for HAR with Hartree–Fock and for DFT with the BLYP functional). In that work it was also observed that cc-pVTZ leads to better ADP values. In summary, switching from cc-pVDZ to the larger basis set cc-pVTZ seems to improve the ADP values. In the case of one of the bonds in this study it also significantly improved the bond length, but it was unclear if this was an isolated case or if such improvement can be expected in certain situations.

#### 3.1.2. Quantum chemistry method   

The following methods were compared: DFT with B3LYP and the BLYP functional, Hartree–Fock(HF), and the post-Hartree–Fock methods: MP2 and CCSD. The same set of quantum chemistry methods was tested in refinements for l-alanine (Wieduwilt *et al.*, 2020 ▸); however, effects of the crystal field were not taken into account in that work. We can roughly order the accuracy of the methods for calculating the energies and corresponding properties in the following way: HF < MP2 ≤ CCSD and BLYP < B3LYP, also MP2 ≃ B3LYP. A general perception of the accuracy of the quantum chemistry method is reflected in the values of the discrepancy factor *wR*_2_, where HF gave the highest values, greater than B3LYP by ≤0.51 p.p. Similar trends were observed in the work by Wieduwilt *et al.* (2020 ▸); however, in the current work MP2, CCSD and B3LYP gave similar agreement factors (measured as *wR*_2_) whereas in the other study CCSD gave superior results. The higher *wR*_2_ values do not automatically translate into higher discrepancies between X-ray and neutron structural parameters (*e.g.* HF produces relatively good bond lengths in this work).

It has been observed that the choice of quantum chemistry method in HAR has a systematic effect on the lengths of bonds to hydrogen. For example, Hartree–Fock of Gly-l-Ala produces systematically too long and BLYP too-short bonds (Capelli *et al.*, 2014 ▸). Too-short N—H bonds (up to 36 mÅ) for refinement with BLYP were also reported for carbamazepine (Sovago *et al.*, 2016 ▸). We have also observed some trends in bond lengths. For all bonds, they can be ordered in the following way (average X-ray value minus the neutron value in mÅ given in parentheses): BLYP (−12.5) < B3LYP (−8) < CCSD (−3.6) ≤ HF (4.2). The same ordering was also observed for l-alanine (Wieduwilt *et al.*, 2020 ▸) for all polar bonds to hydrogen (in the –NH_3_^+^ group). Although the differences are sometimes within experimental uncertainty, the fact that the order of the bond lengths can be observed for all bonds [Fig. 3 ▸(*a*)] suggests that this is not an artefact but a real trend in the bond length values. Some of the differences are quite substantial, the largest one, between BLYP and HF, takes, on average, a value of 17 mÅ.

In terms of bond length accuracy (see 〈|Δ*R*|〉 in Table 1 ▸), BLYP produces the largest discrepancies in bond lengths [12 (6) mÅ on average], B3LYP smaller [8(5) mÅ] and Hartree–Fock and post-Hartree–Fock methods the smallest (3.5–4.6 mÅ). Different conclusions on relative accuracy could be drawn from results for Gly-l-Ala (Capelli *et al.*, 2014 ▸), where the discrepancies for polar bonds for BLYP and Hartree–Fock were quite similar (on average 11 versus 14 mÅ). However, those results are not in contrast with the observation that post-Hartree–Fock methods produce relatively good bond lengths and that B3LYP produces better bond lengths than BLYP. Certainly more tests are required to establish relative accuracy of bond length estimation with various quantum chemistry methods, but it can already be concluded that BLYP leads to too-short bond lengths.

Clear assessment of relative accuracy is also not possible for ADP determination. Some of the worst values of ADP accuracy descriptors are associated with the HF method [Figs. 3 ▸(*b*) and 3(*c*)], which also gave the worst ADP in terms of 〈|Δ*U*_*ij*_|〉 and 〈*S*_12_〉 (Table 1 ▸). Also for Gly-l-Ala (Capelli *et al.*, 2014 ▸) HF-derived ADPs were worse than those from BLYP in terms of 〈|Δ*U*_*ij*_|〉. However, the evidence for the inferiority of the ADP values from HF calculations in the current work is not strong, hence it is probably not possible to draw such a conclusion on the basis of visual inspection of 〈|Δ*U*_*ij*_|〉 for individual atoms [Fig. 3 ▸(*b*)]. In the case of *S*_12_ [Fig. 3 ▸(*c*)] there is one atom with a much higher *S*_12_ value for HF than for other methods (*S*_12_ = 8.7 versus <3) – in oxalic acid, H1 which participates in a very strong hydrogen bond. Interestingly, the large 〈|Δ*U*_*ij*_|〉 in urea from HF (0.0078 for HF versus 0.0044 Å^2^ for MP2) does not translate into a visibly larger wRMSD (1.9 versus 1.7, see Table S1). This is caused by the fact that standard deviations for HF-derived *U*_*ij*_ are about 50% larger than those for MP2 (although they are quite similar in the case of oxalic acid). No trends in the volumes of thermal ellipsoids have been observed [Fig. 3 ▸(*d*)].

#### 3.1.3. Representation of molecular environment   

The most common approach applied in HAR uses point multipoles. It is clear that the lack of such representation leads to an inferior structural model in terms of the averaged discrepancies in both bond lengths and ADP values, and a larger *wR*_2_ for the tested systems (see Table 1 ▸). For all hydrogen atoms, ADP value agreement factors 〈|Δ*U*_*ij*_|〉 and *S*_12_ for models with crystal field representations (CFR) were close to or better than those for models with no such representation [Figs. 4 ▸(*b*) and 4(*c*)]. Volumes of thermal ellipsoids were larger for models with no CFR [Fig. 4 ▸(*d*)]. For bond lengths, the largest discrepancies were also generated with models with no CFR [Fig. 4 ▸(*a*)]. Significant effects for neglecting strong intermolecular interactions were also observed in HAR refinements with fragmentation (fragHAR) for polypeptides (Bergmann *et al.*, 2020 ▸).

Typically in HAR only the molecules/ions constituting the asymmetric unit (hereafter referred as the ‘central part’) are treated at a quantum mechanical level. It was reported (Fugel *et al.*, 2018 ▸) that the accuracy of HAR may improve when molecules/ions surrounding the central part are treated at the quantum mechanical level. We have tested two variants of this approach. In one, quantum mechanical calculations were performed on a cluster of molecules including the central part (urea or oxalic acid with two neighbouring water molecules) and molecules involved in hydrogen bonds with the central part, which lead to a cluster of 56 atoms in the case of urea, and 58 atoms in the case of oxalic acid. This variant is referred to as ‘qm cluster smaller’ in Fig. 4 ▸. Another variant involved those molecules which are up to 3.5 Å from the central part. Corresponding clusters included 88 atoms in the case of urea and 136 atoms for oxalic acid. This variant is referred to as ‘qm cluster larger’ in Fig. 4 ▸. The clusters were also surrounded by point multipoles (using an 8 Å threshold). Replacing the point-charge values and dipoles with explicit quantum mechanical representations of the surrounding molecules did not generate a visible improvement, but significantly increased the computational cost of refinement. It was suggested (Capelli *et al.*, 2014 ▸) that such a representation could improve the accuracy of polar N—H bond lengths and its lack could be a reason why that accuracy was lower than for C—H bonds. Our results do not support such a supposition; however, it seems quite probable that an explicit quantum mechanical representation of neighbouring molecules would be advantageous for systems with very strong interactions. Oxalic acid dihydrate has a very strong hydrogen bond (O1—H1⋯O3), but it was always treated at the quantum mechanical level in this work. This is related to the technical aspects of the implementation (all components of the asymmetric unit have to be represented in the same quantum mechanical calculations).

#### 3.1.4. Combination of less expensive HAR settings   

In this study, we have also examined a combination of settings which are computationally less expensive than B3LYP/cc-pVTZ with surrounding multipoles, which is used as a reference model in this paragraph. We have also performed TAAM refinement with the *UBDB* data bank (Volkov *et al.*, 2007 ▸; Dominiak *et al.*, 2007 ▸; Jarzembska & Dominiak, 2012 ▸; Kumar *et al.*, 2019 ▸) using a locally modified version of *OLEX2*. The reference model clearly gave a better agreement factor than the computationally less expensive models (*wR*_2_, see Table 1 ▸). It also outperformed them in terms of accuracy for ADP values in terms of 〈|Δ*U*_*ij*_|〉 and *S*_12_ (see Table 1 ▸), those values are also consistently relatively low for the method for all hydrogen atoms [see Figs. 5 ▸(*b*) and 5(*c*)].

Interestingly the combination of the quantum chemistry method which led to the highest *wR*_2_ – Hartree–Fock – with the smallest basis set tested – cc-pVDZ – led to the best bond lengths. This seems to be the result of two systematic effects: the Hartree–Fock method giving slightly too-long bonds combined with a smaller basis set which, in the case of Hartree–Fock, leads to shortening of the bond (which can be observed for all bonds with polar hydrogen atoms, see Fig. S2 of the supporting information). Similarly in the work by Capelli *et al.* (2014 ▸) it was observed that the smaller basis set does not lead to inferior bond lengths compared with the larger one for the Hartree–Fock method and average bond lengths are smaller for this basis set.

In the case of urea, lower quality HAR models gave results comparable to TAAM(*UBDB*) [worse in the case of HAR with HF/cc-pVDZ(−), see Table S1]. In the case of oxalic acid dehydrate, with very strong hydrogen bonds, TAAM(*UBDB*) gave clearly worse results [see Table S1 and Fig. 5 ▸(*a*)]. We have also included structural models based on the standardized neutron bond lengths in the comparison. They are in relatively good agreement with those from neutron experiments except for the H1 atom in oxalic acid involved in a very strong hydrogen bond, for which the discrepancy reaches 54 mÅ (which is still less than 73 mÅ in the case of TAAM).

### 3.2. Electron density partition   

In terms of *wR*_2_ statistics, the differences between the partitions are quite small (see Table 2 ▸), maximally 0.07 p.p., which was much less than between models using the cc-pVTZ and cc-pVDZ basis sets (maximum 0.3 p.p.) or between the HF and B3LYP methods (maximum 0.51 p.p.) or – to a lesser extent – between the models with and without surrounding multipoles (up to 0.15 p.p.). The differences were relatively small despite quite large differences in the atomic charge values (see Table 3 ▸), *e.g.* IH partition gave a charge of 0.53 on the oxalic acid H1 atom while the H partition gave 0.12, which means that for H partition the atoms carry almost twice as many electrons as for IH partition (0.88 e versus 0.47 e). Usually the absolute values of the charge for IH, IS and MBIS are significantly larger than those for H and B in the case of polar hydrogen atoms.

Standard uncertainties (SU) for bond lengths (σ_bond_) and ADP values (σ_ADP_) varied significantly between partitions (see Table 4 ▸). The highest values of σ_bond_ for the covalent bonds to hydrogen were observed for refinements with iterative Hirshfeld partition. A similar situation was found for the hydrogen ADP values. The smallest SU values were obtained for the B partition. Those differences were more pronounced for the polar hydrogen atoms (*i.e.* in urea and oxalic acid), *e.g.* the average σ_bond_ in oxalic acid is 8.6 mÅ for IH and 2.7 mÅ for B. For SPAnPS (the non-polar hydrogens), those values were 3.6 and 2.6 mÅ, respectively.

**Figure 6:**
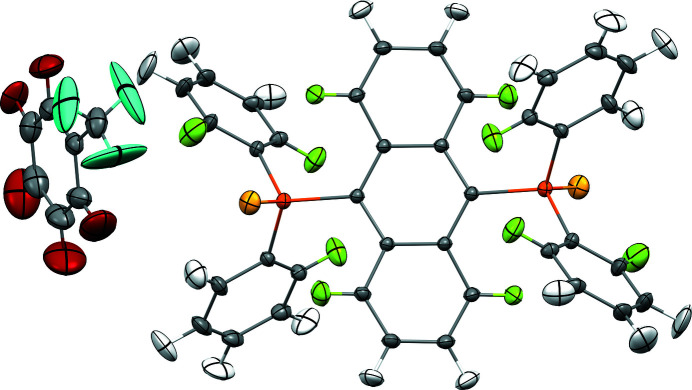
Hydrogen atoms in SPAnPS coloured according to the group (see text): (1) – green, (2) – white, (3) – dark red and (4) – cyan. Iterative Hirshfeld partition refinement with B3LYP/cc-pVTZ theory level and surrounding multipoles cluster. ADP values are shown at the 50% probability level (Mercury, Macrae *et al.*, 2020 ▸).

In the case of SPAnPS, larger discrepancies were observed for atoms with larger ADP values and/or those bonded to carbon atoms with larger ADP values and/or with higher contributions from anharmonic terms in their atomic displacement descriptions. This effect is shown in Table 5 ▸ for Hirshfeld partitions (see Table S2 for data for all partitions) in which the statistics for four groups of hydrogen atoms in SPAnPS are presented (see Fig. 6 ▸): (1) bonded to the carbon atoms for which no anharmonic motion was refined in the work by Köhler *et al.* (2019 ▸), (2) other atoms in the larger molecule, (3) aryl hydrogens in toluene and (4) methyl hydrogens in toluene. There was a very clear increase in 〈|Δ*R*|〉 for subsequent groups; when hydrogen atoms and their bonding partners had larger ADP values and the anharmonic displacement effects were more pronounced, 〈|Δ*R*|〉 was larger. Similar patterns could be observed for 〈|Δ*U*_*ij*_|〉, which was similar for the two groups of hydrogen atoms present in the larger molecule and much larger for the hydrogen atoms in toluene, especially in the methyl group. The larger absolute error in ADP values and bond lengths for atoms with larger ADP values was an expected observation. The more interesting one, however, is a clear increase of the ratio of X-ray to neutron bond lengths (see *R*_X_/*R*_N_ in Table 5 ▸), which rise in the following way: 0.995, 0.998, 1.000, 1.025. One of the possible reasons could be an effect introduced by convolution approximation which is specific to X-ray models. The SPAnPS example suggests that, in general, the discrepancies resulting from large ADP values and anharmonic effects could be much larger than those related to electron density partitions.

Only atoms of group (1) were used for further analysis in SPAnPS (see Table 6 ▸) in order to avoid additional discrepancies between X-ray and neutron results which can be expected for atoms with possibly higher contributions from anharmonic effects and/or with large ADP values. Standard uncertainties for the bond lengths for this group are 2.8–4.6 mÅ for X-ray and 1–1.5 mÅ for neutron data. In this case, 〈|Δ*R*|〉 for all partitions are very similar, about 5 mÅ, and the differences in 〈|Δ*R*|〉 between the partitions did not exceed 1 mÅ and were smaller than the population standard deviations (∼3 mÅ), indicating that all partitions gave C—H bond lengths of similar accuracy for that structure. While wRMSD values in the 1.2–1.56 range did not give a clear indication that there was a statistical difference between the X-ray and neutron bond lengths, all GAR-derived bonds were shorter than the neutron ones suggesting that this is a systematic effect.

**Figure 7:**
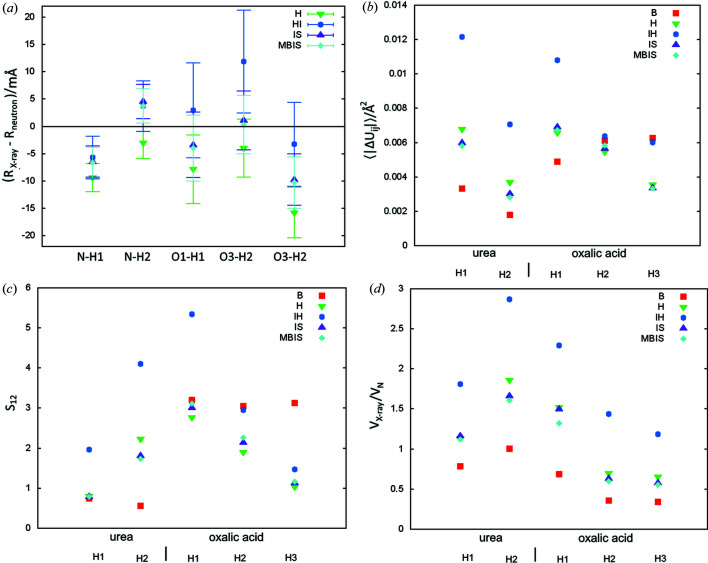
Comparison of the neutron and X-ray parameters of polar hydrogen atoms for refinement with various electron density partitions: (*a*) Δ*R* (mÅ) – the difference between X-ray and neutron measured bond lengths (error bars correspond to the X-ray bond length uncertainties), Fig. S2 also includes B partition, (*b*) 〈|Δ*U*_*ij*_|〉 – average absolute difference of ADP tensor components, (*c*) ADP similarity index *S*_12_, (*d*) *V*_X_/*V*_N_ ratio of X-ray and neutron thermal ellipsoids.

The situation is quite different in the case of urea and oxalic acid, which have only polar hydrogen atoms (see Tables 2 ▸ and 7 ▸). Systematic differences between the partitions could be observed. In all cases the bond lengths can be ordered in the following way: B < H < IS, MBIS, IH [see Fig. 7(*a*) ▸]. IS produced similar bond lengths to MBIS. For N—H bonds (urea) HI also gave similar bond lengths except for O—H (oxalic acid), which were all longer by about 5 mÅ than for IS. The differences in the average bond length between the X-ray and neutron measurements for those bonds were B −18.2, H −8.3, IH 1.7, IS −3.1 and MBIS −3.6 mÅ (see Table 7 ▸).

In terms of bond length, the accuracy for polar hydrogen atoms in MBIS, IS and IH are very similar [4.4 (13), 5.0 (33) and 5.5 (37) mÅ], H is slightly worse [8(5) mÅ]. The differences between MBIS, IS and IH appear to be too small relative to the spread of the results (measured as population standard deviations, see Table 7 ▸) to conclude on the superiority of any of the methods in bond length estimation. H partition probably produces worse results, but more research is needed to justify this statement since the difference between the methods is not large compared with the error spread for each method (see Table 7 ▸) and standard uncertainties of bond lengths. We can clearly point to B as an inferior partition in terms of Δ*R* (see Fig. S3 and Table 7 ▸). Interestingly, it is comparable to other methods in the case of urea but much worse in the case of oxalic acid.

Reported values of wRMSD provide information on the differences relative to its standard deviations. The value for the Hirshfeld partition (2.1) borders the value for which we can conclude that the bond lengths for this partition are statistically different from those obtained from neutron diffraction, in this case they are shorter.

In the case of ADP values, a clear trend could be observed for the volumes of thermal ellipsoids (see the 〈*V*_X_/*V*_N_〉 statistics in Table 2 ▸). For all individual hydrogen atoms in all of the tested systems, the volumes can be ordered in the following way: B < H, IS, MBIS < IH. This regular difference is especially striking in the case of the polar hydrogen atoms [see Fig. 7 ▸(*d*)]. Iterative Hirshfeld refinement produces worse ADP values then the other methods, in terms of both 〈|Δ*U*_*ij*_|〉 and *S*_12_ statistics [see Figs. 7 ▸(*b*) and 7(*c*)]. ADP values from IH are too large, whereas B ADP values tend to be too small (see 〈*V*_X_/*V*_N_〉 values in Table 2 ▸). Data for IH and H are good examples of the situation when values with higher accuracy and precision, in this case for H in terms of |Δ*U*_*ij*_| (Table 2 ▸ and 7) and σ_*U*_*ij*__ (Table 4 ▸), can at the same time correspond to higher wRMSD values (Table 2 ▸).

With B producing inferior bond lengths, IH inferior ADP values and H probably overly short bond lengths, MBIS and IS seem to be the most promising methods. When comparing the partition methods we should bear in mind that the values are reported for a particular method of computational chemistry – DFT with B3LYP functional – and could differ for other methods. Recommendations for GAR settings should be given for a set of settings (*e.g.* IH with B3LYP/cc-pVTZ and point multipoles for crystal field representation) rather than for each setting separately (*e.g.* recommendation for IH without specifying the other settings). In principle, finding such an optimal set of settings would require an exhaustive search over all combinations of the settings. In practice we can try to identify the most promising combinations without exhaustive search. For example, IH produces too-large ADP values for B3LYP and replacing B3LYP with other quantum chemistry methods probably cannot solve this problem since for these methods no systematic trends were observed for thermal ellipsoids volumes. Similarly in the case of B partition. The most promising partitions are therefore H, IS and MBIS. In the case of quantum chemistry methods we can probably eliminate BLYP as it seems to produce too-short bonds and there is probably no partition among the ‘promising’ ones that can correct for that. Also Hartree–Fock would not be a good first choice since it produces clearly inferior agreement factors and this cannot be changed with a different partition, since partitions have a very small effect on the agreement factors. Therefore in the case of quantum chemistry methods B3LYP and post-Hartree–Fock methods seem to be the most promising. Hartree–Fock is also certainly worth further testing as it gave relatively accurate bond lengths in this work despite high *wR*_2_ agreement factor values.

From a practical point of view, refinements with IH partitions turned out to be the slowest to converge in terms of the number of wavefunction calculations required. For SPAnPS and urea, we observed an oscillatory behaviour of some structural parameters when comparing the results of consecutive least square refinements. To account for this, we averaged the structural parameters from the last two least squares refinements and averaged the atomic form factors from the last two calculations of the wavefunction, which led to significantly improved convergence (see Fig. S5).

## 4. Conclusions   

HAR is a refinement technique that allows for the determination of accurate structural parameters for hydrogen atoms from X-ray diffraction data. A generalization of HAR (GAR) was introduced in this work. Aside from the Hirshfeld electron density partition used in the original version of HAR, other partitions were applied: Becke, Hirshfeld, iterative Hirshfeld, iterative stockholder and the minimal basis iterative stockholder. The effects of the electron density partitioning choice on GAR-like refinement were tested on the structures of two small polar organic molecules (urea and oxalic acid dihydrate) and a larger one (SPAnPS) with no polar hydrogen atoms. The effects of partition choice were also compared with those caused by other settings of GAR such as (1) the quantum chemistry method (Hartree–Fock, DFT with BLYP and DFT with B3LYP functional, CCSD, MP2), (2) the basis set, (3) representation of the crystal field and (4) some combination of these factors. Since the set of tested structures is rather small, the results should be treated as suggestive of the most promising HAR-like refinement settings as well as an indication of the most promising directions for a further search for the optimal choice of such settings, and not as a concluding recommendation. In all of the GAR refinements the hydrogen atom positions and anisotropic displacement parameters were refined freely and these refinements led to positively defined ADP tensors. The discrepancies between GAR and neutron-derived bond lengths to hydrogen atoms were much smaller than those which can be expected for the classical model (IAM) with spherical electron densities – the maximal average difference for GAR refinement was 26 mÅ and minimal 3.3 mÅ, compared with about 100 mÅ which can be expected for IAM. In terms of the *wR*_2_ agreement factor, the differences caused by the electron density partitioning scheme were much smaller than those introduced by the choice of basis set (*e.g.* cc-pVDZ versus cc-pVTZ), the method of wavefunction calculation (*e.g.* HF versus B3LYP), or the difference between the refinements with and without crystal field representation (in the case of polar molecules). Therefore none of the partitions were clearly the best in terms of experimental data reconstruction. In the case of the structural parameters, the differences between the partitions were comparable to those caused by changing the other settings. Since the refinement results depend on a combination of GAR settings, it is possible to asses such a combination and not an individual component of the GAR model. In principle, finding such an optimal combination of the settings would require an exhaustive search over all the possible combinations of the settings.

Our analysis does not clearly show which combinations of setting should be recommended for GAR. Among the tested partitions the most promising ones for the further search of optimal settings are Hirshfeld partition, iterative stockholder and minimal basis iterative stockholder. Among the quantum chemistry methods DFT with BLYP functional seems to be the least likely to be used in an optimal combination of settings.

We have analyzed structural parameters related to hydrogen atoms. Many systematic effects related to choice of GAR settings were observed, especially in the case of polar hydrogen atoms. For example, when comparing the results of the refinements using different partitions, in all of the cases bond lengths can be ordered in the following way: Becke < Hirshfeld < other partitions. A clear trend can be also observed in the thermal ellipsoid volumes – Becke partition, for all of the atoms in all of the tested structures, produces the lowest values whereas the iterative Hirshfeld produces the largest values. The effects of a particular partition on the refinement could be summarized as follows (when used with the B3LYP functional and cc-pVTZ basis set):

· The Becke partition was expected to produce inferior results as it was designed purely for the purpose of numerical integration used in density functional theory calculations. It indeed gave the worst results in terms of *wR*_2_ (still a relatively small difference) and seemed to be more prone to produce inferior results than the other partitions. For polar hydrogens, it led to the shortest bonds and the smallest ADP values. However, it gave the smallest standard uncertainties for the hydrogen parameters.

· The Hirshfeld partition gave relatively good ADP values, *wR*_2_, and slightly too-short bond lengths (8.3 mÅ on average)

· Iterative Hirshfeld –gave the largest bond lengths for the polar hydrogens, on average 1.7 mÅ longer than those from neutron measurement. It also gave the largest ADP values, which led to the largest discrepancies in terms of absolute difference when compared with the neutron diffraction results. It also led to the highest standard uncertainties and was more prone to problems with convergence of refinement.

· The iterative stockholder and minimal basis iterative stockholder gave similar results. Bonds to polar hydrogen atoms were on average longer than those from the Hirshfeld partition and slightly more similar to those from the neutron diffraction measurements and the ADP values were relatively good. They seem to produce a slightly better result than other partitions but more research is needed to verify this observation.

Tests of the other factors influencing the HAR-like refinement performed on the crystal structures of urea and oxalic acid showed also some systematic trends (all refinements were performed with Hirshfeld partition of the electron density):

· Similarly to the previous publications regarding the classical HAR approach, the cc-pVDZ basis set was clearly inferior to cc-pVTZ in terms of *wR*_2_ but extending the basis set further to cc-pVQZ did not give a clear improvement. The cc-pVTZ basis set gave systematically larger volumes for thermal ellipsoids than cc-pVDZ. For oxalic acid it also led to clearly better structural parameters.

· Bonds to hydrogen atoms derived using GAR with the Hartree–Fock method were systematically shorter when the cc-pVDZ basis set was used instead of cc-pVTZ (by 6 mÅ on averge).

· Systematic differences in bond lengths calculated with different quantum chemistry methods have been observed for polar hydrogen atoms. They can be ordered in the following way for averaged X-ray–neutron values (given in parentheses in mÅ): BLYP(−12) < B3LYP(−8) < CCSD(−4) < HF(4).

· Hartree–Fock was clearly an inferior method for wavefunction calculation in terms of *wR*_2_. B3LYP seemed to be a better choice than BLYP in terms of bond length accuracy and agreement factors, and the application of the most expensive methods (MP2, CCSD) gave similar agreement factors as B3LYP but with better bond lengths. Yet when B3LYP was paired with other electron density partitions – *i.e.* the iterative stockholder – the bond length accuracy improved and was similar to the accuracy of MP2 and CCSD with the Hirshfeld partition.

· While there is a clear advantage in representing the crystal field with multipoles, we did not observe further improvement when treating the surrounding molecules quantum mechanically (such an improvement was expected for compounds with very strong intermolecular interactions). Refinement with no crystal field representation led to the largest volumes of thermal ellipsoids in the case of polar hydrogen atoms.

· TAAM, which is based on the use of fixed, predefined parameters derived from the Hansen–Coppens multipole model, performed roughly similarly to HAR with B3LYP/cc-pVDZ and no crystal field representation with point multipoles or with HF/cc-pVDZ and point multipoles in the case of urea. It was however clearly worse than any of the HAR approaches in the case of oxalic acid, which is a system with very strong hydrogen bonds.

Those results may differ when other partition methods are used rather than the applied Hirshfeld, especially in the case of quantum chemistry methods which also exhibit systematic differences in bond lengths.

While it is becoming clear that GAR (including HAR) is probably the most accurate method for deriving structural parameters for hydrogen atoms from X-ray refinement, it is still unclear what the optimal method settings are. With this work we add another dimension to the methodology. The next step will be to test various combinations of the settings using a larger set of test structures and a wider choice of quantum chemistry methods and electron density partitions. Further improvement towards ultra-accurate X-ray refinements may also require a more advanced model of atomic displacements and an examination of the effects of the convolution approximation.

## Supplementary Material

Supporting figures and tables. DOI: 10.1107/S2052252520013603/lt5030sup1.pdf

Supporting cifs. DOI: 10.1107/S2052252520013603/lt5030sup2.zip

CCDC reference: 2040066

## Figures and Tables

**Table 1:** *wR*_2_ statistics and the comparison of structural indicators related to hydrogen atoms (based on comparison with neutron data) for the various settings of HAR, for TAAM and for structures with standardized bond lengths (see text), the averages for five polar hydrogen atoms (in urea and oxalic acid) The symbols (+) and (−) indicate a model with and without point multipoles, respectively. 〈|Δ*R*|〉 – the average absolute difference of bond lengths, 〈Δ*R*〉 – the average difference of the bond lengths, wRMSD(Δ*R*) – the weighted root mean squared deviation for bond lengths [equation (2)], *S*_12_ – the average ADP similarity index *S*_12_ [equation (4)], 〈|Δ*U*_*ij*_|〉 – the average absolute difference of ADP tensor components, wRMSD(Δ*U*_*ij*_) – the weighted root mean squared deviation for the components of the ADP tensor [equation (3)]. Values in brackets correspond to population standard deviations.

	*wR*_2_ oxalic urea acid		〈|Δ*R*|〉 (mÅ)	〈Δ*R*〉 (mÅ)	wRMSD(Δ*R*)	*S*_12_	〈|Δ*U*_ij_|〉 × 10^4^(Å^2^)	wRMSD (Δ*U*_*ij*_)
Basis set								
cc-pVDZ	4.01	1.98	11.2 (78)	−11.2 (78)	2.13	2.9 (14)	58 (14)	1.61
cc-pVTZ	3.73	1.68	8.0 (46)	−8.0 (46)	1.94	1.75 (74)	52 (14)	1.58
cc-pVQZ	3.71	1.69	7.2 (49)	−7.2(4.9)	1.76	1.7 (6)	49 (12)	1.55
Method								
HF	4.01	2.19	4.6 (36)	4.2 (41)	1.06	3.4 (27)	61 (20)	1.65
BLYP	3.78	1.78	12.5 (57)	−12.5 (57)	2.88	2.0 (8)	57 (16)	1.62
B3LYP	3.73	1.68	8.0 (46)	−8.0 (46)	1.94	1.75 (74)	52 (14)	1.58
MP2	3.72	1.66	3.5 (28)	−1.9 (40)	0.91	1.8 (8)	49 (14)	1.53
CCSD	3.73	1.71	3.8 (38)	−3.6 (39)	1.00	1.8 (9)	50 (13)	1.57
Environment								
(−)	3.87	2.07	12.0 (9)	−11.0 (10)	2.77	2.6 (15)	69 (26)	1.74
(+)	3.73	1.68	8.0 (46)	−8.0 (46)	1.94	1.75 (74)	52 (14)	1.58
Cluster hydrogen-bond	3.71	1.65	8.8 (47)	−8.8 (47)	1.85	1.9 (7)	54 (14)	1.60
Cluster 3.5 Å	3.70	1.70	8.2	−7.5 (60)	1.92	1.8 (6)	52 (13)	1.59
Mix of less accurate settings								
B3LYP/cc-pVTZ(+)	3.73	1.68	8.0 (46)	−8.0 (46)	1.94	1.75 (74)	52 (14)	1.58
B3LYP/cc-pVDZ(−)	4.16	2.18	17.0 (7)	−15.0 (11)	3.09	3.4 (10)	70 (25)	1.72
HF/cc-pVDZ(+)	4.17	2.44	3.3 (16)	−1.5 (33)	0.71	5.0 (3)	68 (17)	1.71
HF/cc-pVDZ(−)	4.35	2.51	15.0 (5)	−6.6 (140)	2.75	5.0 (2)	82 (33)	1.77
TAAM (UBDB)	5.15	2.13	38.0 (27)	−38.0 (27)	5.52	7.6 (36)	106 (48)	1.95
Standard bond distance	–	–	18.0 (18)	−13.0 (22)	–	–	–	

**Table 2:** *R* factors (*R*1 and *wR*_2_) and structural indicators related to hydrogen atoms (based on comparison to neutron data) for various electron density partitions in GAR B – Becke, H – Hirshfeld, IH – iterative Hirshfeld, IS – iterative stockholder, MBIS – minimal basis iterative stockholder. 〈|Δ*R*|〉 – average absolute difference of bond lengths, wRMSD(Δ*R*) - weighted room mean square deviation for bond lengths [equation (2)], *R*_X_/*R*_N_ – average ratio of X-ray to neutron bond length, 〈|Δ*U*_*ij*_|〉 – average absolute difference of ADP tensor components, wRMSD(Δ*U*_*ij*_) – weighted room mean square deviation for components of ADP tensor [equation (3)], *S*_12_ – average ADP similarity index *S*_12_ [equation (4)], 〈*V*_X_/*V*_N_〉 – average ratio of X-ray to neutron volumes of thermal ellipsoids.

	*R*1	*wR*2	〈|Δ*R*|〉 (mÅ)	wRSMD (Δ*R*)	*R*_X_/*R*_N_	〈|Δ*U*_*ij*_|〉 × 10^4^	wRSMD (Δ*U*_*ij*_)	*S*_12_	〈*V*_X_/*V*_N_〉
Oxalic acid									
B	1.38	3.79	26.8	6.8	0.973	58	1.8	3.13	0.46
H	1.36	3.73	9.2	1.8	0.990	52	1.4	1.90	0.95
IH	1.37	3.73	6	0.8	1.003	77	1.4	3.26	1.64
IS	1.36	3.72	4.7	1.1	0.996	53	1.4	2.09	0.90
MBIS	1.36	3.73	4.9	1.1	0.995	53	1.5	2.18	0.83
Urea									
B	1.37	1.70	5.6	2.8	0.995	26	1.42	0.65	0.89
H	1.36	1.68	6.2	2.1	0.994	52	1.83	1.52	1.49
IH	1.37	1.72	4.7	1.1	0.999	96	2.06	3.04	2.34
IS	1.37	1.72	5.5	1.6	0.999	45	1.69	1.30	1.41
MBIS	1.38	1.73	5.2	1.5	0.999	43	1.67	1.26	1.36
SPAnPS									
B	2.22	2.54	13.0	4.76	1.006	103	3.74	3.20	0.78
H	2.19	2.47	12.1	3.71	1.002	139	3.72	4.48	1.03
IH	2.20	2.49	11.4	3.41	1.002	154	3.71	4.86	1.07
IS	2.19	2.48	12.2	3.97	1.004	123	3.56	4.02	0.95
MBIS	2.19	2.48	12.4	4.04	1.003	120	3.58	4.07	0.90

**Table 3:** Atomic charges of hydrogen atoms in oxalic acid and urea for various electron density partitions B – Becke, H – Hirshfeld, IH – iterative Hirshfeld, IS – iterative stockholder, MBIS – minimal basis iterative stockholder. For the atom labelling scheme, see Fig. 1 ▸.

	Oxalic Acid			Urea	
	H1	H2	H3	H1	H2
B	0.171	0.167	0.148	0.167	0.170
H	0.119	0.232	0.217	0.153	0.163
IH	0.503	0.589	0.572	0.492	0.504
IS	0.529	0.541	0.523	0.458	0.460
MBIS	0.535	0.554	0.537	0.464	0.465

**Table 4:** Average standard deviations for bond lengths to hydrogen (×10^−4^ Å) and hydrogen *U*_*ij*_ (×10^−4^ Å^2^) for various electron density partitions B – Becke, H – Hirshfeld, IH – iterative Hirshfeld, IS – iterative stockholder, MBIS – minimal basis iterative stockholder) taken from B3LYP/cc-pVTZ refinements with surrounding charges and dipoles. Statistics for SPAnPS based on group 1 hydrogen atoms

	Molecule	B	H	HI	IS	MBIS
〈σ_bonds_〉	Urea	17	27	43	30	30
	OXZDH	27	54	86	54	54
	SPAnPS	26	35	36	33	32
						
〈σ_*U*_*ij*__〉	Urea	10	15	23	16	15
	OXADH	16	27	43	26	25
	SPAnPS	20	25	26	23	22

**Table 5:** Comparison of the structural indicators related to hydrogen atoms with neutron measurements for SPAnPS Hydrogen atoms were divided into groups (see text); results for HAR. 〈|Δ*R*|〉 – average absolute difference of bond lengths, wRMSD(Δ*R*) – weighted room mean square deviation for bond lengths [equation (2)], *R*_X_/*R*_N_ – average ratio of X-ray to neutron bond length, 〈|Δ*U*_*ij*_|〉 – average absolute difference of ADP tensor components, wRMSD(Δ*U*_*ij*_) – weighted room mean square deviation for components of ADP tensor [equation (3)], *S*_12_ – average ADP similarity index *S*_12_ [equation (4)], 〈*V*_X_/*V*_N_〉 – average ratio of X-ray to neutron volumes of thermal ellipsoids.

Hydrogen atoms group	〈|Δ*R*|〉 (mÅ)	wRSMD (Δ*R*)	*R*_X_/*R*_N_	〈|Δ*U_ij_*|〉 × 10^4^	wRSMD (Δ*U*_*ij*_)	*S*_12_	〈*V*_X_/*V*_N_〉
1	5.1 (37)	1.33	0.995	49 (9)	2.19	1.26 (50)	1.04
2	7.9	2.17	0.998	45	1.36	1.05	0.94
3	15.2	4.27	1.000	163	2.22	3.97	1.11
4	31.4	5.68	1.025	530	3.15	21.01	1.14

**Table 6:** Comparison of the structural parameters related to hydrogen atoms with neutron measurements of SPAnPS for group 1 (see text) 〈|Δ*R*|〉 – average absolute difference of bond lengths, wRMSD(Δ*R*) – weighted room mean square deviation for bond lengths [equation (2)], 〈*R*_X_/*R*_N_〉 – average ratio of X-ray to neutron bond lengths, 〈|Δ*U*_*ij*_|〉 – average absolute difference of ADP tensor components, wRMSD(Δ*U*_*ij*_) – weighted room mean square deviation for components of ADP tensor [equation (3)], *S*_12_ – average ADP similarity index *S*_12_ [equation (4)], 〈*V*_X_/*V*_N_〉 – average ratio of X-ray to neutron volumes of thermal ellipsoids.

	〈|Δ*R*|〉 (mÅ)	wRSMD (Δ*R*)	*R*_X_/*R*_N_	〈|Δ*U_ij_*|〉 × 10^4^	wRSMD (Δ*U*_*ij*_)	*S*_12_	〈*V*_X_/*V*_N_〉
B	4.3 (33)	1.49	0.996	36 (4)	2.21	0.97 (33)	0.80
H	5.1 (37)	1.33	0.995	49 (9)	2.19	1.26 (50)	1.04
IH	4.5 (36)	1.20	0.996	52 (10)	2.22	1.31 (51)	1.07
IS	5.0 (32)	1.48	0.995	41 (7)	2.11	1.05 (45)	0.96
MBIS	5.2 (27)	1.56	0.995	38 (6)	2.04	0.97 (44)	0.92

**Table 7:** Comparison of structural parameters related to hydrogen atoms with neutron measurements for various electron density partitions (electron density form B3LYP/cc-pVTZ with crystal field represented with point multipoles): average values for five polar hydrogen atoms (in urea and oxalic acid) 〈Δ*R*〉 – average difference of bond lengths, 〈|Δ*R*|〉 – average absolute difference of bond lengths, wRMSD(Δ*R*) – weighted room mean square deviation for bond lengths [equation (2)], *R*_X_/*R*_N_ – average ratio of X-ray to neutron bond lengths, 〈|Δ*U*_*ij*_|〉 – average absolute difference of ADP tensor components, wRMSD(Δ*U*_*ij*_) – weighted room mean square deviation for components of ADP tensor [equation (3)], *S*_12_ – average ADP similarity index *S*_12_ [equation (4)], 〈*V*_X_/*V*_N_〉 – average ratio of X-ray to neutron volumes of thermal ellipsoids. Partition acronyms: B – Becke, H – Hirshfeld, IH – iterative Hirshfeld, IS – iterative stockholder, MBIS – minimal basis iterative stockholder.

	〈Δ*R*〉 (mÅ)	〈|Δ*R*|〉 (mÅ)	wRSMD (Δ*R*)	*R*_X_/*R*_N_	〈|Δ*U*_ij_|〉 × 10^4^	wRSMD (Δ*U*_*ij*_)	*S*_12_	〈*V*_X_/*V*_N_〉
B	-18 (13)	18 (13)	5.7	0.982 (13)	45 (19)	1.90	2.2 (14)	0.6 (3)
H	-8(5)	8(5)	2.1	0.992 (5)	52 (15)	1.55	1.7 (8)	1.2 (5)
IH	2(7)	5.5 (37)	1.1	1.001 (7)	85 (28)	1.53	3.2 (16)	1.9 (7)
IS	-3(6)	5.0 (33)	1.5	0.997 (6)	50 (17)	1.55	1.8 (9)	1.1 (5)
MBIS	-3(6)	4.4 (13)	1.3	0.997 (6)	49 (19)	1.42	1.8 (9)	1.0 (5)
