# Supplementary material for: Hirshfeld atom like refinement with alternative electron density partitions
Source: IUCrJ. 2020 Oct 29;7(Pt 6):1199–215. doi: 10.1107/S2052252520013603 (PMC7642787; doi:10.1107/S2052252520013603)
Supplement: Supplementary file 1 [file m-07-01199-sup1.pdf]

IUCrJ

Volume 8 (2021)

Supporting information for article:

Hirshfeld atom like refinement with alternative electron density partitions

Michał Leszek Chodkiewicz, Magdalena Woińska and Krzysztof Woźniak

Table S1 wR_2 statistics and comparison of structural indicators related to hydrogen atoms (compared to neutron data) for various settings of HAR and for TAAM and structures with standardized bond lengths (see text). (+): indicates model with point multipoles, (-): without point multipoles. $\langle|\Delta R|\rangle$ - average absolute difference of bond lengths, $\langle S_{12}\rangle$ – average ADP similarity index S_{12} (Eq. 4), $\langle|\Delta U_{ij}|\rangle$ average absolute difference of ADP tensor components, wRMSD(ΔU_{ij}) - weighted room mean square deviation for components of ADP tensor (Eq. (3)).

	wR_2		$\langle|\Delta R|\rangle$(mÅ)		$\langle S_{12}\rangle$ /$\langle|\Delta U_{ij}|\rangle$ x 10^4/wRMSD	
system	Oxalic acid	Urea	Oxalic acid	Urea	Oxalic acid	Urea
**Basis set**						
cc-pVDZ	4.01	1.98	15.9	4.1	3.93/59/1.6	1.40/64/1.6
cc-pVTZ	3.73	1.68	9.2	6.2	1.90/52/1.4	1.52/52/1.8
cc-pVQZ	3.71	1.69	8.7	4.8	1.74/48/1.4	1.59/49/1.8
**Method**						
HF	4.01	2.19	4.6	4.5	4.06/50/1.4	2.50/78/1.9
BLYP	3.78	1.78	13.6	10.7	2.07/57/1.4	2.01/57/1.8
B3LYP	3.73	1.68	9.2	6.2	1.90/52/1.4	1.52/52/1.8
MP2	3.72	1.66	4.1	2.7	2.33/51/1.4	1.12/44/1.7
CCSD	3.73	1.71	5.3	1.4	2.20/49/1.4	1.32/52/1.8
**Environment**						
(-)	3.87	2.07	14.8	8.8	2.22/60/1.4	3.38/80/2.1
(+)	3.73	1.68	9.2	6.2	1.90/52/1.4	1.52/52/1.8
Cluster H-bond	3.71	1.65	11.1	5.2	1.88/51/1.4	1.93/58/1.8
Cluster 3.5 Å	3.70	1.70	10.1	5.1	1.93/51/1.4	1.56/54/1.9
**Mix of less accurate settings**						
B3LYP/cc-pVTZ(+)	3.73	1.68	9.2	6.2	1.90/52/1.4	1.52/52/1.8
B3LYP/cc-pVDZ(-)	4.16	2.18	22.7	9.6	3.50/61/1.4	3.22/84/2.0
HF/cc-pVDZ(+)	4.17	2.44	2.7	4.1	6.30/62/1.7	3.09/78/1.8
HF/cc-pVDZ(-)	4.35	2.51	13.8	15.8	4.67/61/1.5	5.39/114/2.1
TAAM (UBDB)	5.15	2.13	59.7	6.9	10.30/130	3.46/69/1.9
Standard bond dist.	-	-	26	6.5	-	-

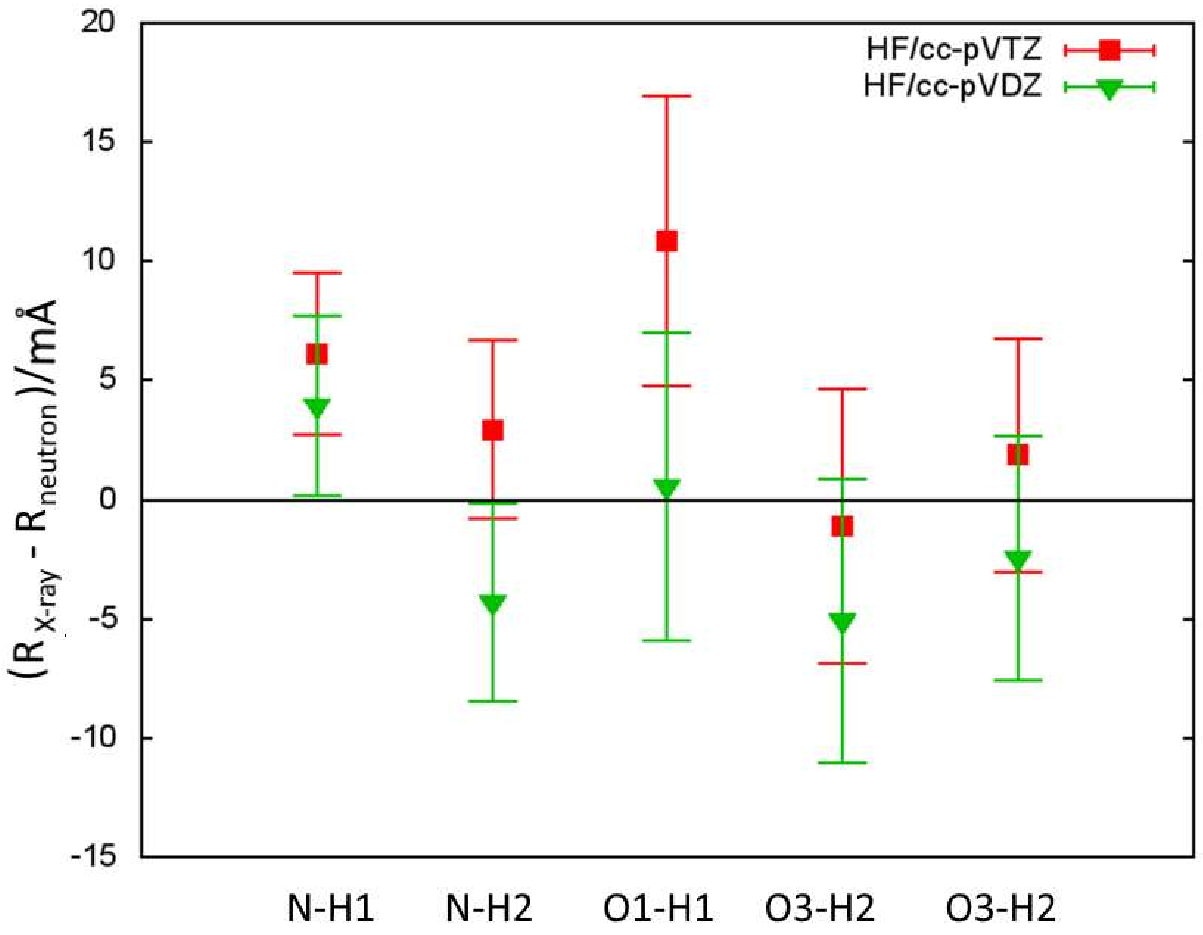

Figure S1 The difference between HAR and neutron measured bond lengths (error bars corresponds to HAR bond lengths uncertainties) for Hartree-Fock method with cc-pVDZ and with cc-pVTZ basis set.

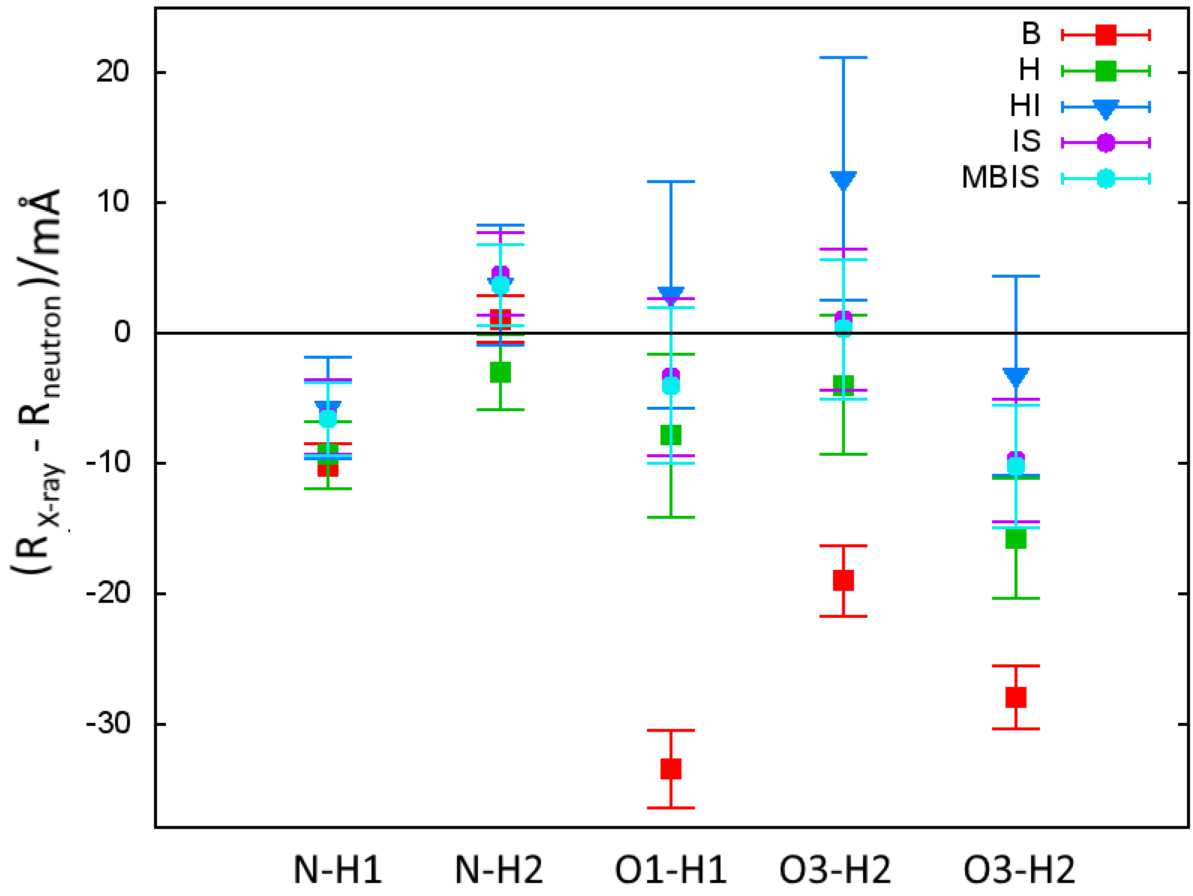

Figure S2 The difference between X-ray and neutron measured bond length in mÅ (error bars corresponds to X-ray bond lengths uncertainties).

Table S2 *C*omparison of the structural parameters related to hydrogen atoms with neutron measurements for SPAnPS. Differences between the neutron and GAR structural parameters were small relative to the corresponding standard uncertainties and did not clearly indicate which partition methods gave the most accurate results. H atoms were divided into groups (see text).

	⟨|ΔR|⟩ (mÅ)	wRSMD (ΔR)	$\langle R_X/R_N \rangle$	⟨|ΔU_{ij}|⟩ x 10^4	wRSMD (ΔU_{ij})	S_{12}	⟨V_X/V_N⟩
H atoms, group (1)							
B	4.3(33)	1.49	0.996	36(4)	2.21	0.97(33)	0.80
H	5.1(37)	1.33	0.995	49(9)	2.19	1.26(50)	1.04
IH	4.5(36)	1.20	0.996	52(10)	2.22	1.31(51)	1.07
IS	5.0(32)	1.48	0.995	41(7)	2.11	1.05(45)	0.96
MBIS	5.2(27)	1.56	0.995	38(6)	2.04	0.97(44)	0.92
H atoms, group (2)							
B	6.7	2.48	1.000	41	1.48	1.16	0.68
H	7.9	2.17	0.998	45	1.36	1.05	0.94
IH	7.6	2.04	0.999	48	1.38	1.09	0.97
IS	7.2	2.34	0.999	41	1.33	0.95	0.85
MBIS	7.3	2.36	0.999	40	1.36	0.97	0.81
H atoms, group (3) (aryl toluene atoms)							
B	14.3	4.64	1.006	102	1.98	1.80	0.81
H	15.2	4.27	1.000	163	2.22	3.97	1.11
IH	14.6	4.14	1.000	173	2.22	4.11	1.16
IS	14.1	5.20	1.002	134	2.11	3.20	0.98
MBIS	14.6	5.37	1.002	131	2.10	3.28	0.96
H atoms, group (4) (toluene methyl group)							
B	44.1	9.29	1.041	401	3.26	15.36	1.00
H	31.4	5.68	1.025	530	3.15	21.01	1.14
IH	29.4	5.32	1.025	613	3.10	23.33	1.22
IS	35.9	6.52	1.033	487	3.15	19.49	1.15
MBIS	36.0	6.57	1.033	479	3.16	19.86	1.04

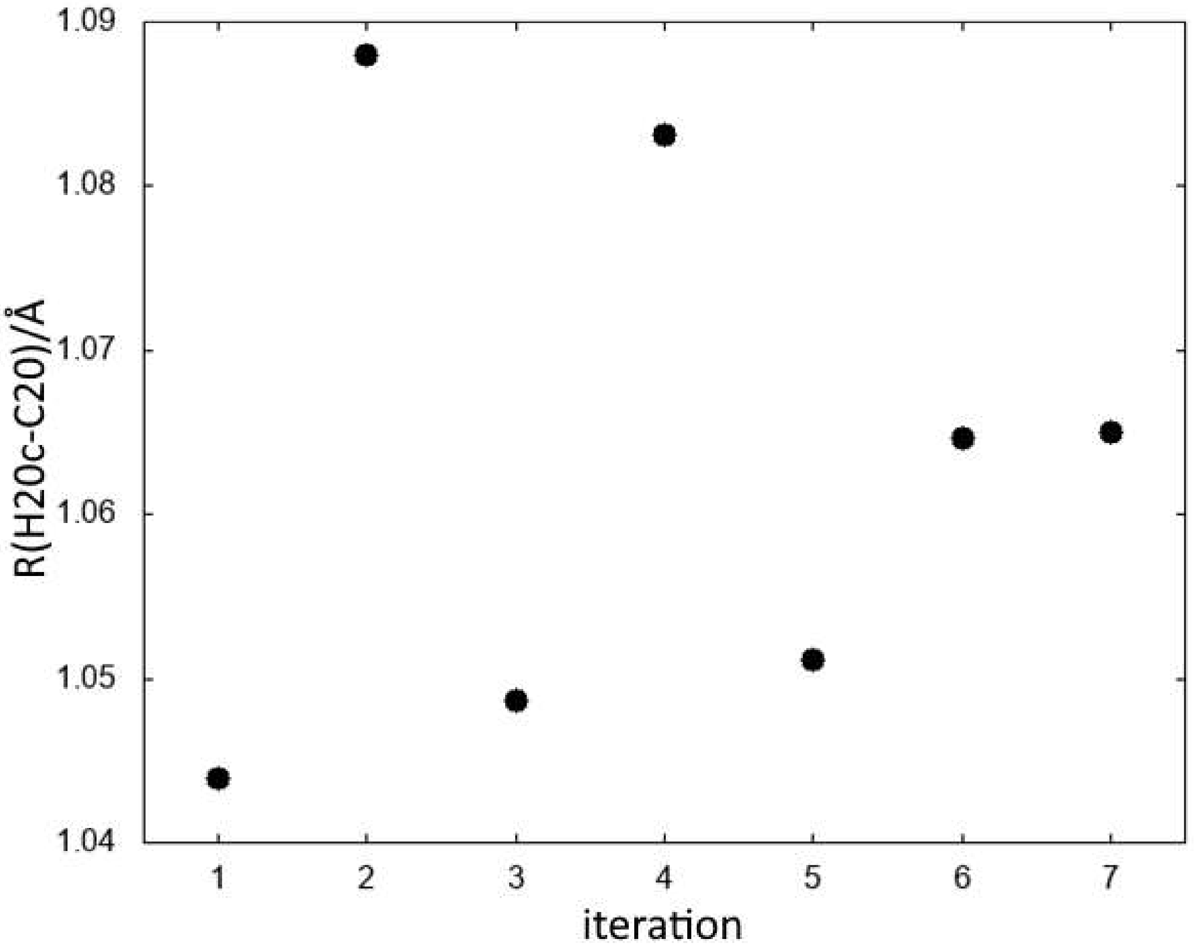

Figure S3 Convergence of H20c-C20 bond length in SPAnPS. This is the bond in the toluene methyl group which length showed oscillatory behavior in refinement causing problems with convergence of GAR refinement with IH partition. After the 5th iteration, an averaging procedure (see text) was applied.
